# Construction of a lncRNA-associated competing endogenous RNA regulatory network after traumatic brain injury in mouse

**DOI:** 10.1186/s13041-022-00925-8

**Published:** 2022-05-02

**Authors:** Siqi Wang, Yiyu Sun, Shaobo Hu, Cen Lou, Yuan-Bo Pan

**Affiliations:** 1 Department of Nuclear Medicine, Sir Run Run Shaw Hospital, Zhejiang University School of Medicine, Hangzhou, 310000 Zhejiang China; 2 Department of Neurosurgery, Second Affiliated Hospital, School of Medicine, Zhejiang University, NO. 88 Jiefang Rd, Hangzhou, 310009 Zhejiang China; 3 Department of Plastic and Reconstructive Surgery, Shanghai 9th People’s Hospital, Shanghai Jiao Tong University, 639 Zhi Zao Ju Road, Shanghai, 200011 China; 4 Department of Neurosurgery, Li Hui Li Hospital of Medical Center of Ningbo, Ningbo, Zhejiang China

**Keywords:** Bioinformatics analysis, Traumatic brain injury, ceRNA, lncRNA, Neat1, Myd88

## Abstract

Traumatic brain injury (TBI) is a major public health problem worldwide which causes high mortality and disability. Functioning as microRNA (miRNA) sponges, long non-coding RNA (lncRNA) regulates the expression of protein-coding genes in a competing endogenous RNA (ceRNA) network. However, the lncRNA-associated ceRNA in TBI remains unclear. In this study, we processed the raw SRR files of mice cortex samples of sham injury (n = 3) and TBI groups (n = 3) to count files. Then, the expression profiles of lncRNAs and mRNAs were identified, and 86 differentially expressed (DE) lncRNAs and 1201 DEmRNAs between sham and TBI groups were identified. The DEmRNAs were used to perform enrichment analyses. Next, a lncRNA-miRNA-mRNA regulatory ceRNA network was constructed. The network consisted of 23 mRNAs, 5 miRNAs and 2 lncRNAs. The expression alternations of the 5 miRNAs were validated via qRT-PCR. The subnetwork of hub lncRNA Neat1 was extracted. We identified a potential inflammatory associated regulatory axis: Neat1/miR-31-5p/Myd88 axis. The PPI network based on DEmRNA involved in ceRNA network was constructed PPI networks to identify the hub genes. Finally, DElncRNAs and DEmRNAs were selected randomly and validated by qRT-PCR. In conclusion, with the lncRNA-miRNA-mRNA ceRNA network provided above, we can improve our understanding of the regulatory mechanisms and interaction among lncRNAs, miRNAs and mRNAs in TBI process.

**Supplementary Information:**

The online version contains supplementary material available at 10.1186/s13041-022-00925-8.

## Introduction

Traumatic brain injury (TBI) is a major public health problem all over the world. It is estimated that 10 million people worldwide suffer from TBI annually [1, 2]. WHO estimates that TBI will be the third most common cause of death and disability in global terms by 2020 [3]. TBI can be divided into initial injury and secondary brain injury [4]. Initial injury causes cells to die in the moment of mechanical shock. Secondary injury refers to the biochemical and physiological changes which would cause the apoptosis and death of neural cells, the formation of brain edema, the disruption of the blood–brain barrier, and the subsequent neurological disorders [5]. Various efforts have been made over the past decades, however, TBI remains a disease with high mortality and disability, which brings a huge burden on the families and society [6]. The complex molecular mechanisms behind brain injury need to be further explained.

Long non-coding RNAs (lncRNAs) belong to the non-coding RNA family with more than 200 nucleotides in length [7]. Although lncRNAs do not encode any protein products, many studies have proved that they could be involved in the regulation of gene expression at epigenetic, transcriptional or post-transcriptional levels [8, 9]. Several studies have shown that lncRNAs can act as miRNA sponges in the ceRNA regulatory network and further regulate protein expression ^10^. In recent years, more and more studies have focused on the role of lncRNA-related ceRNA regulatory networks in intracranial aneurysm [11], cerebral infarction [12], type 2 diabetes [13] and various types of cancer [14, 15, 16]. Several lncRNAs, such as lncRNA GM12371, lncRNA Evf2 and lncRNA Pinky, were reported to be involved in regulation of synaptic functions and neurodevelopment [17, 18, 19]. Moreover, some lncRNAs were found to be specifically expressed in nervous system, such as cerebellar cortex [20, 21]. These findings indicated that lncRNAs could serve as a major role in the nervous system. However, the potential role of the lncRNA-associated ceRNA regulatory network in TBI remains unclear.

In this study, we investigated the expression of lncRNAs and mRNAs in the injury cortex 24 h after TBI in mice via RNA-seq data analyses and further constructed a lncRNA-miRNA-mRNA ceRNA regulatory network. Firstly, we identified differentially expressed lncRNAs (DElncRNA) and mRNAs (DEmRNAs) between normal brain cortex and injury cortex. The DEmRNAs were used to perform enrichment analyses. Then, we developed a lncRNA-miRNA-mRNA ceRNA regulatory network using integrated bioinformatics analysis. The network contained 23 mRNAs, 5 miRNAs and 2 lncRNAs. The expression alternations of the 5 miRNAs were validated via qRT-PCR. Next, Subnetwork of Neat1 was further analyzed, and inflammatory related Neat1/miR-31-5p/Myd88 axis was identified. Gene Oncology and Kyoto Encyclopedia of Genes and Genomes (KEGG) pathway enrichment analyses were conducted to evaluate the mRNAs in the network to identify additional biological functions. Furthermore, protein–protein interaction (PPI) network was constructed, and hub genes with key roles in the PPI network were identified. Finally, the difference in expression of four DElncRNA and four DEmRNA was validated via qRT-PCR. The construction of the lncRNA-miRNA-mRNA ceRNA network may provide insight into the regulatory mechanism of TBI.

## Materials and methods

### Animals

All animal procedures were performed in accordance with the Guidelines for Care and Use of Laboratory Animals of the Zhejiang University and approved by the Animal Ethics Committee of the Zhejiang University. Adult male C57BL/6 mice were purchased from Shanghai Slac Experimental Animal Center.

### Controlled cortical impact (CCI) model of TBI

Both sham-injury and TBI mice (12–16 weeks old and 22–25 g) were anesthetized by intraperitoneal injection of 1% pentobarbital sodium solution (0.1 ml/20 g). Six mice were divided into sham group (n = 3) and TBI group (n = 3) according to the random number table. The mice heads were shaved and disinfected by wiping with iodophors. The animals were mounted in a prone position on a stereotaxic instrument (RWD Life Science, China) and fixed with auxiliary ear and incisor bars. Using the sterile surgical procedures, the mice received a midline cranial skin incision, and the scalp was retracted to expose the skull. Then, the mice received a right lateral craniotomy (3.5 mm in diameter) with 1.5 mm lateral to sagittal suture and 2.0 mm posterior to the bregma remaining the dura mater intact, which was performed with a motorized drill. The cortical impact was performed at a velocity of 5.0 m/s, a depth of 2.0 mm below the cortical surface, and an impact duration of 180 ms, which would cause a moderately severe contusion in the sensorimotor cortex. Medical grade cyanoacrylate gel was applied to the exposed dura mater and skull surface after CCI. The hole on the skull was filled with bone wax, and the skin incision was sutured with an absorbable suture. The antibiotic ointment was applied to the suture area. The mice were wrapped in an electric blanket to maintain the body temperature and transferred to a clean cage. Sham injury mice underwent the same craniotomy and postoperative care procedures. All the mice were anesthetized with 1% pentobarbital sodium solution (0.1 ml/20 g) 24 h post-TBI. The mice were transcardially perfused with 10 mL 4℃ 0.9% saline. The ipsilateral cortex around the contusion site was dissected rapidly and stored at − 80℃. All the 6 cortex samples were used for qRT-PCR.

### RNA-seq data processing

The SRR files (SRR3271216-SRR3271221) of mice cortex samples of sham injury (n = 3) and TBI groups (n = 3) were retrieved from Gene Expression Omnibus (GEO) NCBI [22], which were converted to ‘fastq’ format data using sratoolkit (version 2.9.6). Using FastQC (version 0.11.9), quality control of the raw sequence data was performed. Low-quality reads and adaptors were removed by the trimmomatic software (version 0.36). The reference genome and genome annotation files of mouse (Release M24 GRCm38.p6) were downloaded from GENCODE (www.gencodegenes.org). Then, the high-quality reads were aligned to the reference genome with hisat2 (version 2.0.0) [23]. The generated SAM (Sequence Alignment/Map) files were converted to the BAM (Binary Alignment/Map) format by using samtools (version 1.10) [24]. The BAM files were converted to counts files by using featureCounts in subread software (version 2.0.0) [25]. Then, the “.csv” files were generated, each consisting of all gene counts for a particular sample. All of the “.csv” files were combined, and a single file with sample names depicted as columns and gene names depicted as rows was obtained. LncRNAs and mRNAs were annotated according to the genome annotation files.

### Identification of differentially expressed lncRNAs and mRNAs

Using the DESeq2 package [26], the counts files were normalized and differentially expressed analyses were performed. lncRNAs and mRNAs with |log2(fold change)|> 1 and adjusted P-values < 0.05 were considered DElncRNAs and DEmRNAs.

### Functional enrichment analysis

Both the Gene ontology (GO) enrichment and ﻿Kyoto Encyclopedia of Genes and Genomes (KEGG) pathway enrichment analyses were performed using the Database for Annotation, Visualization, and Integrated Discovery platform (DAVID 6.8; https://david.ncifcrf.gov/) [27]. DEmRNAs or the mRNAs involved in ceRNA network were used for GO and KEGG pathway enrichment analyses. The GO terms and pathways, with corrected P-values < 0.05 using the Benjamini method, were considered significant functional categories.

### Construction of ceRNA network

Firstly, the interactions of DElncRNAs and targeted miRNAs with high stringency (> = 3) were identified using starBase v2.0 [28]. Then, miRNA-targeted mRNAs were identified using miRTarBase, a highly reliable miRNA reference database [29]. The miRTarBase database has accumulated more than three hundred and sixty thousand miRNA-targeted interactions which were experimentally validated by western blot, reporter assay, microarray and next-generation sequencing experiments. Moreover, the targeted mRNAs that were not differentially expressed between control cortex and TBI cortex samples were filtered out. The ceRNA network was constructed and viewed using Cytoscape ﻿(version 3.7.2; http://www.cytoscape.org/) [30].

### Construction of protein–protein interaction (PPI) network

The Search Tool for the Retrieval of Interacting Genes (STRING version 11.0; www.string-db.org) was used to construct the PPI network of DEmRNAs involved in the ceRNA network [31]. The interactions with a score more than 0.4 were included. The PPI network was constructed and viewed using Cytoscape (version 3.7.2).

### RNA preparation and qRT-PCR

The brain cortex tissues of sham and TBI groups were cut into small chunks and rinsed with PBS, which were further dissolved with TRIzol reagent (Invitrogen, USA) to acquire total RNA. Then, using Reverse Transcription Kit (TaKaRa, Osaka, Japan), the isolated RNA was reverse-transcribed to cDNA. The qRT-PCR analyses were performed on an Applied Biosystems 7500 Fast Real-Time PCR System (Roche, Basel, Switzerland) with SYBR Green supermix (172-5150, Bio-Rad, Shanghai, China). All the experiments were performed and analyzed in triplicate. Five miRNAs (mmu-miR-377-3p, mmu-miR-185-5p, mmu-miR-107-3p, mmu-miR-31-5p, and mmu-miR-130a-3p), four DElncRNAs (lncRNA C030018K13Rik, lncRNA Gm36823, lncRNA H19 and lncRNA Mir155hg) and four DEmRNA (P2ry12, Hes5, Cxcr2 and Mmp12) were randomly selected to perform qRT-PCR for validation. The primer sequences used in this study were shown in Additional file 1. The lncRNA, miRNAs and mRNA expression levels were calculated according to the 2^−ΔΔCt^ method.

### Statistical analysis

GraphPad Prism (version 6.0, GraphPad Software, San Diego, CA, USA) and R language (3.4.3) were used for statistical analysis. Statistical differences were determined by Student’s t test for two-group comparisons. A P-value < 0.05 was considered statistically significant. Barplots were generated by GraphPad Prism. Other plots, including bubble charts, volcano plots and heat maps, were produced by R language (3.4.3).

## Results

### LncRNA expression profile

**Fig. 1:**
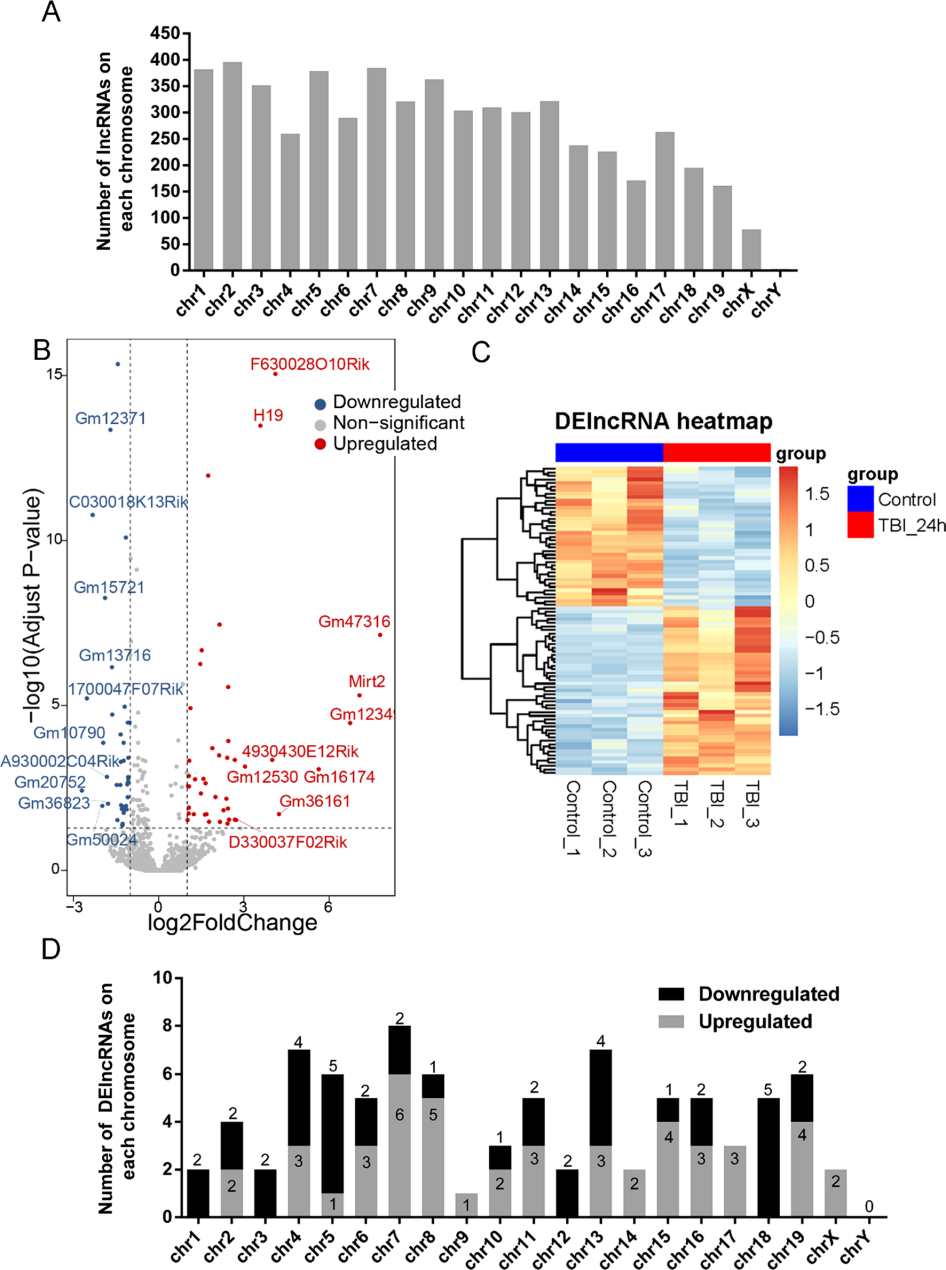
LncRNA profile based on RNA-seq data. **A** Distribution of all identified lncRNAs in TBI samples. **B** Volcano plot of DElncRNAs in mice cortex 24 h post-TBI compared with control cortex. Red points represent upregulated DElncRNAs and blue points represent downregulated DElncRNAs in TBI group. The genesymbols of ten upregulated lncRNAs and ten downregulated lncRNAs with the most significant expression differences are shown in the plot. **C** Hierarchical cluster heatmaps of DElncRNAs. Each row represents an RNA, and each column represents a sample. Red indicates relatively high expression, and blue indicates relatively low expression. **D** Distribution of DElncRNAs in TBI, showing upregulated (gray) and downregulated (black) lncRNAs in each chromosome (chr)

The total detected reads for samples from sham (n = 3) and TBI (n = 3) groups were 100,290,212 and 96,819,024, respectively. The RNA-seq analysis of 3 normal brain cortex tissues and 3 TBI cortex tissues identified 9945 lncRNAs. After deleting lncRNAs with average read count below 1 across all samples, there were 5700 lncRNAs remaining. The distribution of these lncRNAs in the chromosomes was shown in Fig. 1A. Of these lncRNAs, 86 lncRNAs were differentially expressed (|log_2_FC|> 1 and adjusted P-value < 0.05) in mouse cortex 24 h post-TBI relative to normal brain cortex, which contained 47 upregulated lncRNAs and 39 downregulated lncRNAs (Fig. 1B, Additional file 2). Hierarchical clustering analysis showed that lncRNA expression profiles in the injured cortex were significantly different from those in the control group (Fig. 1C). The differentially expressed lncRNAs (DElncRNAs) were distributed on all the chromosomes, although the distribution on the chromosomes was not equal (Fig. 1D). Chromosome 7 had the largest number of DElncRNAs, of which 6 were upregulated and 2 were downregulated, accounting for 9.3% (8/86) of all DElncRNAs.

### mRNA expression profile

**Fig. 2:**
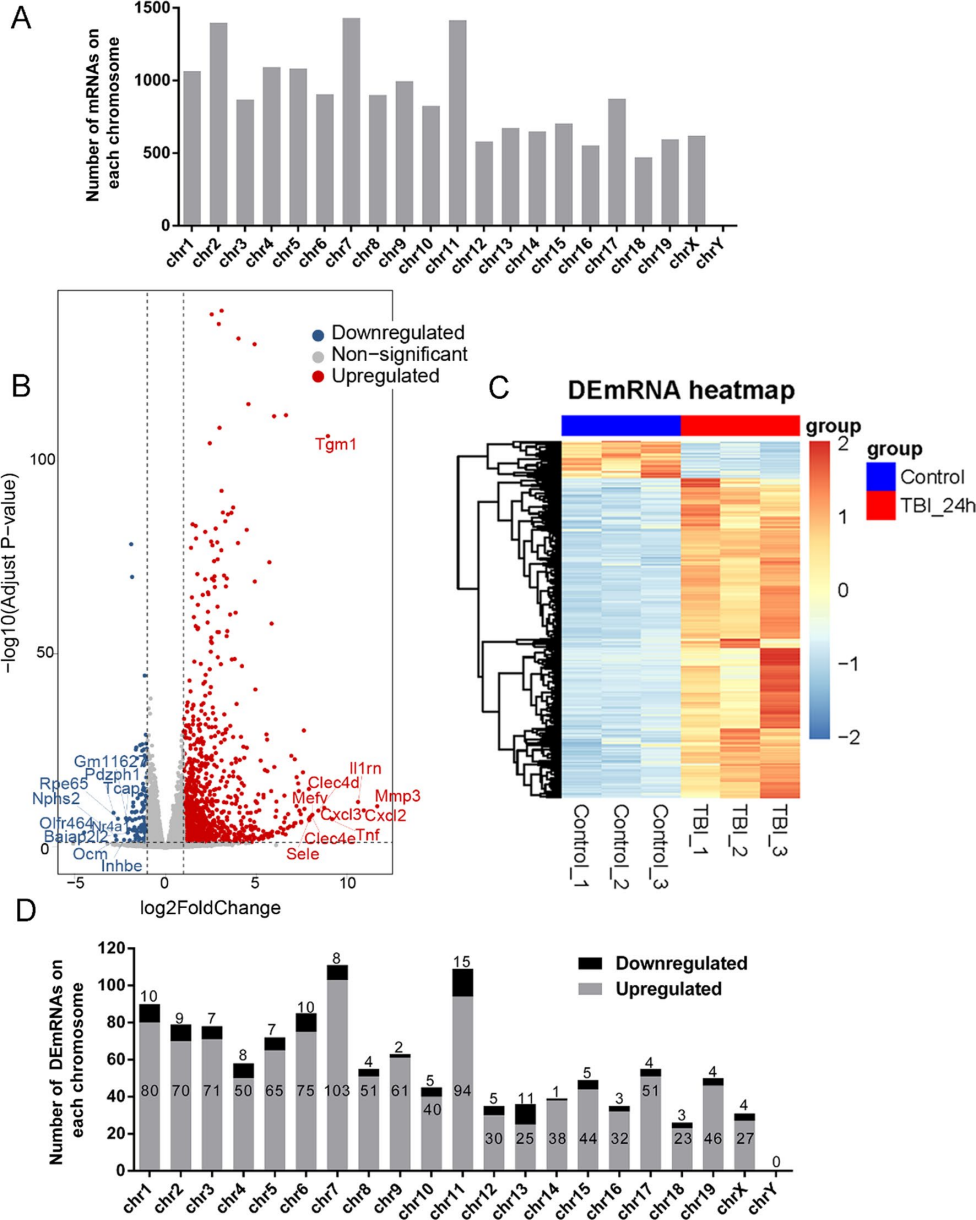
mRNA profile based on RNA-seq data. **A** Distribution of all identified mRNA in TBI samples. **B** Volcano plot of DEmRNAs in mice cortex 24 h post-TBI compared with control cortex. Red points represent upregulated DEmRNAs and blue points represent downregulated DEmRNAs in TBI group. The genesymbols of ten upregulated mRNAs and ten downregulated mRNAs with the most significant expression differences are shown in the plot. **C** Hierarchical cluster heatmaps of DEmRNAs. Each row represents an RNA, and each column represents a sample. Red indicates relatively high expression, and blue indicates relatively low expression. **D** Distribution of DEmRNAs in TBI, showing upregulated (gray) and downregulated (black) mRNAs in each chromosome (chr)

The RNA-seq analysis of 3 normal brain cortex tissues and 3 TBI cortex tissues also identified 21,807 mRNAs. After deleting mRNAs with average read count below 1 across all samples, there were 17,711 mRNAs remaining. The distribution of these mRNAs on all the chromosomes was shown in Fig. 2A. 1201 differentially expressed mRNAs (DEmRNAs) were identified between mouse cortex 24 h post-TBI and normal brain cortex, which contained 1076 upregulated mRNAs and 125 downregulated mRNAs (Fig. 2B, Additional file 2). Hierarchical clustering analysis showed that mRNA expression profiles in the injured cortex were significantly different from those in the normal cortex (Fig. 2C). The DEmRNAs were distributed on all the chromosomes, although the distribution on the chromosomes was not equal (Fig. 2D). Chromosome 7 had the largest number of DEmRNAs, of which 103 were upregulated and 8 were downregulated, accounting for 9.24% (111/1201) of all DEmRNAs.

### Gene ontology and pathway analysis of DEmRNAs

**Fig. 3:**
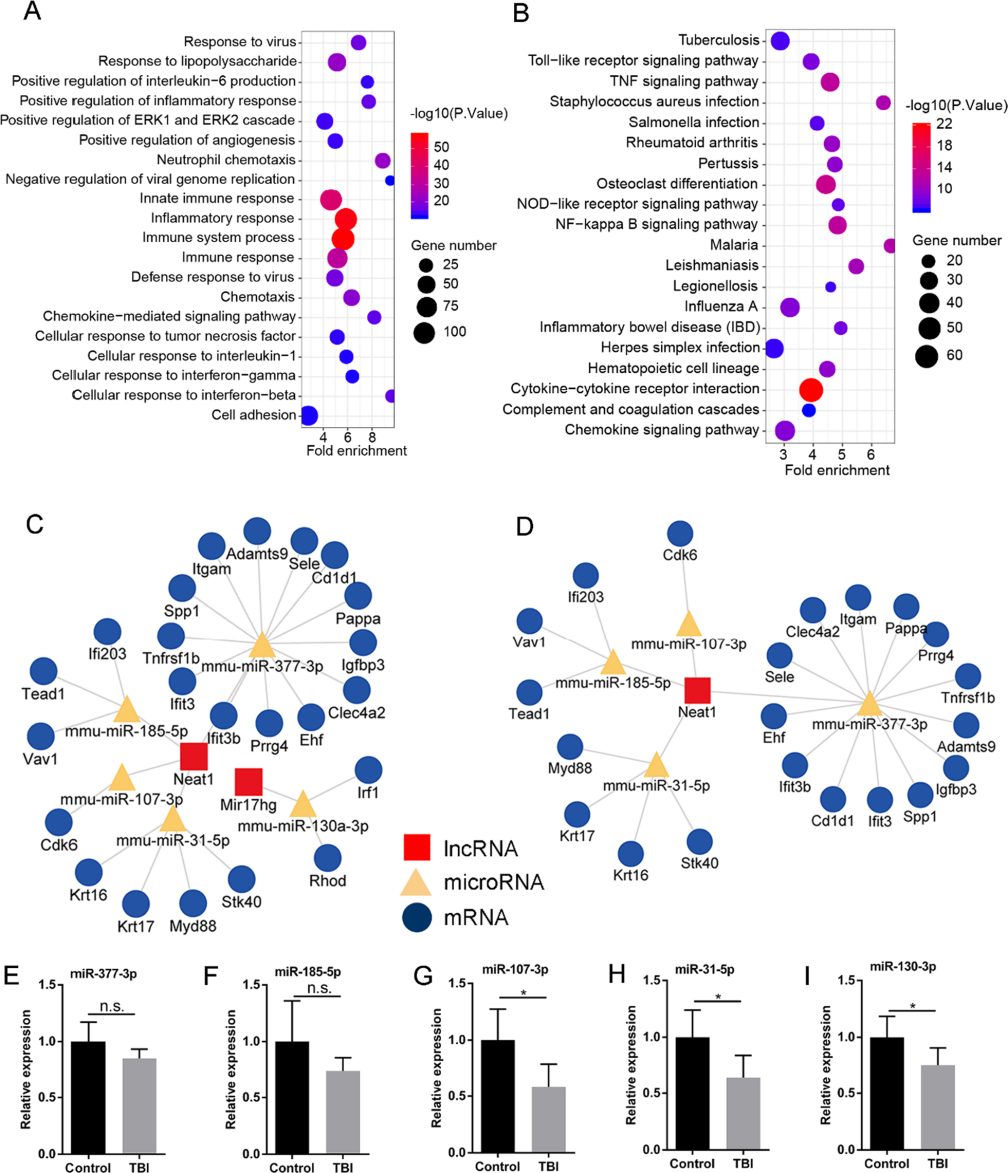
**A** The top 20 enriched terms in the GO biological process analysis. **B** The top 20 enriched pathways in the KEGG pathway analysis. **C**, **D** ceRNA network and subnetwork related to TBI. **C** lncRNA-associated ceRNA regulatory network related to TBI. **D** Subnetwork of lncRNA Neat1. Blue squares represent lncRNAs, tan triangles represent miRNAs, and red circles represent mRNAs. **E**–**I** Validation of expression alternations of miRNAs after TBI via qRT-PCR. All the experiments were performed and analyzed in triplicate. (*P < 0.05)

To explore the potential functional implication of the 1,201 DEmRNAs, GO enrichment and KEGG pathway analyses were performed. In the GO enrichment analysis, a total of 466 enriched GO terms in the Biological Process (BP) were identified. The top 20 significantly enriched terms were shown (Fig. 3A). The DEmRNAs were primarily enriched in immune inflammatory response-related BPs, such as “immune system process”, “inflammatory response”, “neutrophil chemotaxis”, “immune response” and “response to lipopolysaccharide”. Furthermore, we also found that these DEmRNAs were also enriched in “cell response to interferon-beta”, “cellular response to tumor necrosis factor”, “cellular response to interleukin-1” and “cellular response to interferon-gamma”, which indicated that the neural cells may be actively adapting to the drastically changing microenvironment. In addition, a total 62 enriched pathways were identified via the KEGG pathway analysis. The top 20 significantly enriched pathways were shown (Fig. 3B). Among these pathways, “TNF signaling pathway” and “NF-kappa B signaling pathway” were corresponding to the enriched GO terms, which indicated that the two pathways may play significant roles in acute phase of TBI.

### Construction of a ceRNA regulatory network in TBI

To elucidate the regulatory mechanism of TBI, a lncRNA‐miRNA‐mRNA ceRNA network of TBI was developed. As for integrated ceRNA network, lncRNA‐miRNA‐mRNA axis consists of two forms: (1) downregulated lncRNAs, upregulated miRNAs, and downregulated mRNAs; (2) upregulated lncRNAs, downregulated miRNAs, and upregulated mRNAs. According to the changes in the expression of previously reported miRNAs after TBI [32, 33], we included the five miRNAs (mmu-miR-377-3p, mmu-miR-185-5p, mmu-miR-107-3p, mmu-miR-31-5p, and mmu-miR-130a-3p) with the correct expression trends to develop the lncRNA‐miRNA‐mRNA ceRNA network (Fig. 3C). The ceRNA network in this study only contained upregulated lncRNAs, downregulated miRNAs and upregulated mRNAs. The expression levels of mmu-miR-377-3p, mmu-miR-185-5p, mmu-miR-107-3p, mmu-miR-31-5p, and mmu-miR-130a-3p in brain tissues were reported to downregulate after TBI in previous studies [32, 33]. Moreover, we used qRT-PCR to further confirm the expression alternations of the five miRNAs in network (Fig. 3E–I). The lncRNA‐miRNA and miRNA‐mRNA relationship pairs were listed in Additional file 3 and Additional file 4. The network was constructed with 30 nodes (23 mRNAs, 5 miRNAs and 2 lncRNAs) and 28 edges. Blue squares represented lncRNAs, tan triangles represented miRNAs, and red circles represented mRNAs. Among the network, lncRNA Neat1 directly connected with 4 miRNAs and indirectly connected with 21 mRNAs, which indicated that lncRNA Neat1 could play an important role in acute phase of TBI.

Previous studies reported that downregulation of miR-377 could promote angiogenesis and inhibit inflammation to alleviate ischemic brain injury and renal ischemia/reperfusion injury [34, 35]. Feng et al. found that lncRNA ADAMTS9-AS2 could upregulate IGFBP-2 expression via decreasing miR-185-5p expression, further promoting angiogenesis [36]. Moreover, several studies have confirmed that lncRNA Neat1 could sponge miR-185-5p to regulate IGF2 expression or DNMT1/mTOR signaling, promoting cancer progression [37, 38]. In addition, the lncRNA Neat1 was reported to regulate CDK6 and CDK14 via sponging miR107 and further promote tumor growth [39, 40, 41]. Previous study found that over-expressed lncRNA Neat1 could promote axon growth of neurons in vitro, inhibit cell apoptosis and restrict inflammation after TBI [42]. To investigate the downstream mechanism of Neat1, we further analyzed subnetwork of Neat1 (Fig. 3D). For example, in this ceRNA network, an upregulated DEmRNA, myeloid differentiation factor 88 (Myd88), were reported to be overexpressed dramatically in brain tissues after TBI and activate NF-κB as well as promote the production of proinflammatory cytokines [43]. Moreover, Cai et al. found that miR-31-5p/Myd88/ NF-κB pathway plays an important role in blood–brain barrier damage after subarachnoid hemorrhage [44]. In addition, upregulated miR-31-5p was reported to inhibit cell apoptosis [45]. Yang et al. revealed that lncRNA Neat1 was able to modulate inflammatory responses via miR-31-5p/POU2F1 axis [46]. Thus, we suggested that Neat1/miR-31-5p/Myd88 axis may be a crucial pathway in TBI process.

### Functional enrichment analyses of TBI ceRNA network

**Fig. 4:**
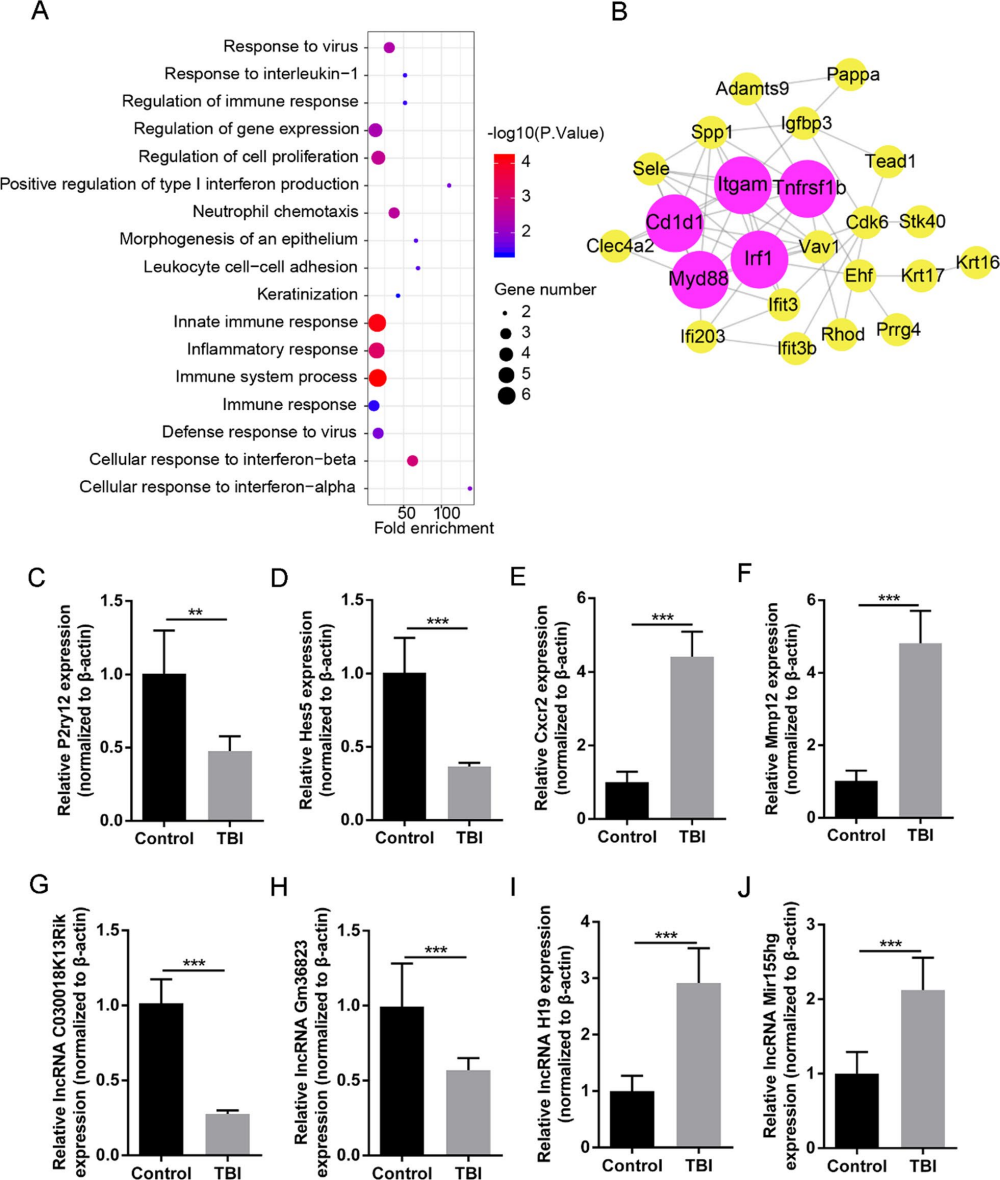
**A** The top 20 enriched terms in the GO biological process analysis. **B** PPI network of mRNAs in ceRNA network. Circles represent protein-coding genes, and edges represent the interactions between two proteins. The purple circles represent top 10 hub genes with highest degrees. **C**–**J** Validation of expression differences of DElncRNAs and DEmRNAs via qRT-PCR. **C**–**D** P2ry12 and Hes5 expression significantly decrease in injury cortex 24 h post-TBI compared to control cortex. **E**–**F** Cxcr2 and Mmp12 expression significantly increase in injury cortex 24 h post-TBI compared to control cortex. **G**–**H** lncRNA C030018K13Rik and Gm36823 expression significantly decrease in injury cortex 24 h post-TBI compared to control cortex. **I**–**J** lncRNA H19 and Mir155hg expression significantly increase in injury cortex 24 h post-TBI compared to control cortex. All the experiments were performed and analyzed in triplicate. (*P < 0.05, **P < 0.01, ***P < 0.001)

To explore the biological functions of ceRNA network in TBI, we performed the GO and KEGG pathway analyses for mRNAs involved in the network. The top 20 enriched GO terms were shown in Fig. 4A. The terms of signaling pathway regulation was significantly enriched, including “positive regulation of I-kappaB kinase/NF-kappaB signaling”, “positive regulation of JNK cascade” and “positive regulation of ERK1 and ERK2 cascade”. The significant enriched terms of “angiogenesis” and “positive regulation of smooth muscle cell proliferation” indicated that the ceRNA network may be involved in blood vessel reconstruction of cortex post-TBI. Moreover, the ceRNA network was also involved in immune and inflammatory response.

### Construction of PPI network and identification of hub genes

To further explore the relationship among the DEmRNA involved in ceRNA network, we constructed PPI networks via the STRING database. The PPI network was shown in Fig. 4B. Furthermore, using Cytoscape App cytoHubba, we identified the hub genes with highest degrees (bigger purple circles), including Myd88, Tnfrsf1b, Cd1d1, Itgam and Irf1. These hub genes could play important roles in TBI ceRNA network.

### Validation of DElncRNAs and DEmRNAs using qRT-PCR

To validate the reliability of the RNA-seq data, we randomly selected two up-regulated lncRNAs (lncRNA H19 and lncRNA Mir155hg), two down-regulated lncRNAs (lncRNA Gm36823 and lncRNA C030018K13Rik), two up-regulated mRNAs (Cxcr2 and Mmp12) and two down-regulated mRNAs (Hes5 and P2ry12) that were abundantly expressed and exhibited significant changes. We used qRT-PCR to analyze expression differences in control and injury cortex. The qRT-PCR analysis results were mostly consistent with the RNA-seq data (Fig. 4C–J).

## Discussion

In recent years, an increasing number of studies have revealed that ncRNAs, including lncRNAs and miRNAs, play a key role in TBI [47, 48]. More and more studies have focused on the role of lncRNA-related ceRNA regulatory networks in various diseases, such as cerebral infarction [12], type 2 diabetes [13] and cardiac hypertrophy [49]. However, the potential role of the lncRNA-associated ceRNA regulatory network in TBI still remains unclear. In the present study, 9945 lncRNAs and 21,807 mRNAs were identified via RNA-seq analysis. 86 lncRNAs (47 upregulated and 39 downregulated) and 1201 mRNAs (1076 upregulated and 125 downregulated) were detected to dysregulated in the cortex of mice 24 h after TBI. Functional enrichment analyses indicated that dysregulated genes were involved in immune inflammatory processes and cell responses to tumor necrosis factor/interferon-gamma/interferon-beta/interleukin-1. Moreover, ceRNA regulatory network was constructed based on 23 mRNAs, 5 miRNAs and 2 lncRNAs. Function prediction indicated that the network was involved in angiogenesis, immune and inflammatory response, and activation of several signaling pathways. The subnetwork of Neat1 was further analyzed. Furthermore, PPI network was constructed and 5 hub genes were identified. Finally, randomly selected DEmRNAs and DElncRNAs were validated via qRT-PCR. This study could provide a comprehensive perspective on the underlying lncRNA regulatory mechanism in TBI.

The upregulation of LncRNA Neat1 was found to function in early apoptosis. Zhong et al. [42] explored the relationship between Neat1 and bexarotene in TBI treatment in mice. They found that bexarotene can upregulated Neat1, which further inhibited apoptosis and inflammation, contributing to better motor and cognitive function after TBI. In this study, we found that 5 genes in Neat1 subnetwork were involved in regulation of apoptosis, such as Igfbp3, Myd88, Itgam, Tnfrsf1b and Spp1 [50, 51, 52]. The potential miRNAs between these apoptosis-related genes and Neat1 were also shown in the subnetwork (Fig. 3D). Besides, we revealed that 5 genes in Neat1 subnetwork were involved in regulation of inflammatory response, such as Myd88, Krt16, Spp1, Tnfrsf1b and Sele. Likewise, the potential miRNAs between these inflammatory response-related genes and Neat1 were also shown in the subnetwork (Fig. 3D). Interestingly, miR-31-5p was reported to be able to regulate the angiogenesis of vascular endothelial cells [53]. In addition, previous study found that miR-31-5p was involved in blood–brain barrier repair and Myd88/NF-κB pathway-mediated inflammation after subarachnoid hemorrhage [44]. Furthermore, Myd88 was significantly upregulated after TBI and was able to activate NF-κB and then regulate inflammation during TBI process [43]. Thus, we suggested that Neat1/miR-31-5p/Myd88 axis might play an important role in regulation inflammation cytokines and blood–brain barrier repair in brain tissues after TBI, which needs to be further confirmed in future study.

The expression of lncRNA H19 in brain tissues was upregulated after intracerebral hemorrhage (ICH), which could activate NF-κB and enhance inflammatory responses to aggravate brain edema and neurological injury [54, 55]. The role of lncRNA Mir155hg in brain injury has rarely been reported. Li et al. reported that upregulation of lncRNA Mir155hg was able to promote M1 phenotype macrophage polarization and the release of proinflammatory cytokines in chronic obstructive pulmonary disease [56]. However, the function of lncRNA Gm36823 and lncRNA C030018K13Rik has not been reported so far. Cxcr2, a chemokine receptor on cellular surface, could upregulate and activate microglia in cerebral stoke, Alzheimer’s disease and multiple sclerosis [57–59, 60]. Moreover, Cxcr2 might be involved in neutrophil infiltration and subsequent neurodegeneration following TBI [61, 62]. The Hes5 was reported to be downregulated after brain injury [63, 64], while the Mmp12 expression was significantly increased in intracerebral hemorrhage [65]. However, the function of Mmp12 and Hes5 in brain injury needs to further studied. P2ry12 as a microglia marker could be utilized to evaluate the microglial cells. Previous study found that P2ry12 positive microglia were significantly increased after TBI [66]. However, in this study, we found the P2ry12 expression was decreased after TBI. The different expression trends might be attributed to injury severity, brain regions or time points for sampling.

To summary, a lncRNA-associated ceRNA regulatory network of TBI was successfully constructed, which may provide a comprehensive view of the underlying mechanisms of gene regulation and interaction in TBI. Moreover, we proposed that the regulatory network centred on lncRNA Neat1 may play a critical part in TBI. Additionally, we further suggested that the Neat1/miR-31-5p/Myd88 axis might be potential downstream molecular bases for Neat1 to regulate apoptosis, inflammation and blood–brain barrier damage. However, these regulatory axes require further studies to confirm the molecular mechanisms.

## Supplementary Information

**Additional file 1.** PCR primes used in this study.

**Additional file 2.** The list of DElncRNAs and DEmRNAs.

**Additional file 3.** Interactions between lncRNA and miRNA in the ceRNA network.

**Additional file 4.** Interactions between miRNA and mRNA in the ceRNA network.

## Data Availability

The datasets used or analyzed during the current study are available from the corresponding author on reasonable request.
