# Correction to: Construction of a lncRNA-associated competing endogenous RNA regulatory network after traumatic brain injury in mouse

**DOI:** 10.1186/s13041-022-00950-7

**Published:** 2022-07-13

**Authors:** Siqi Wang, Yiyu Sun, Shaobo Hu, Cen Lou, Yuan‑Bo Pan

**Affiliations:** 1 Department of Nuclear Medicine, Sir Run Run Shaw Hospital, Zhejiang University School of Medicine, Hangzhou, 310000 Zhejiang China; 2 Department of Plastic and Reconstructive Surgery, Shanghai 9th People’s Hospital, Shanghai Jiao Tong University, 639 Zhi Zao Ju Road, Shanghai, 200011 China; 3 Department of Neurosurgery, Li Hui Li Hospital of Medical Center of Ningbo, Ningbo, Zhejiang China; 4 Department of Neurosurgery, Second Affiliated Hospital, School of Medicine, Zhejiang University, NO. 88 Jiefang Rd, Hangzhou, 310009 Zhejiang China

## Correction to: Molecular Brain (2022) 15:40 10.1186/s13041-022-00925-8

Following publication of the original article [1], the authors identified an error in the affiliations assignment: the affiliation ‘Department of Neurosurgery, Second Affiliated Hospital, School of Medicine, Zhejiang University’ was incorrectly assigned to Yiyu Sun as a second affiliation, while this author is only affiliated to the ‘Department of Plastic and Reconstructive Surgery, Shanghai 9th People’s Hospital, Shanghai Jiao Tong University’.

This error is corrected in the author list of this Correction article and the original article [1] has been updated.
