# Supplementary material for: Construction of a lncRNA-associated competing endogenous RNA regulatory network after traumatic brain injury in mouse
Source: Mol Brain. 2022 May 2;15:40. doi: 10.1186/s13041-022-00925-8 (PMC9063179; doi:10.1186/s13041-022-00925-8)
Supplement: Supplementary file 1 — Additional file 1. PCR primes used in this study. [file 13041_2022_925_MOESM1_ESM.docx]

**Additional file 1. PCR primers used in this study.**

| Gene | Primer (5’→3’) |
| --- | --- |
| P2ry12 | F: CCCTGTGCGTCAGAGACTAC  R: CAAGCTGTTCGTGATGAGCC |
| Hes5 | F: AGTCCCAAGGAGAAAAACCGA  R: GCTGTGTTTCAGGTAGCTGAC |
| Cxcr2 | F: ATGCCCTCTATTCTGCCAGAT  R: GTGCTCCGGTTGTATAAGATGAC |
| Mmp12 | F: GGGCTGCTCCCATGAATGAC  R: CCAGAGTTGAGTTGTCCAGTTG |
| β-actin | F: GTGACGTTGACATCCGTAAAGA  R: GCCGGACTCATCGTACTCC |
| C030018K13Rik | F: TTCTGGCTGGTGTCCTAA  R: TGATCCTCACGCATCTCA |
| Gm36823 | F: AATCATCAGGGACCAAAC  R: GCCGTCCTACTTAGCATC |
| H19 | F: GCCTCGCTCCACTGACCTT  R: CCATCTTGCCCTGTTTGAC |
| Mir155hg | F: TGCTGCAAACCAGGAAGG  R: TGGGCTTGAAGTTGAGATGTT |
