# Supplementary material for: Construction of a lncRNA-associated competing endogenous RNA regulatory network after traumatic brain injury in mouse
Source: Mol Brain. 2022 May 2;15:40. doi: 10.1186/s13041-022-00925-8 (PMC9063179; doi:10.1186/s13041-022-00925-8)
Supplement: Supplementary file 3 — Additional file 3. Interactions between lncRNA and miRNA in the ceRNA network. [file 13041_2022_925_MOESM3_ESM.docx]

**Additional file 3. Interactions between lncRNA and miRNA in the ceRNA network.**

| lncRNA | miRNA |
| --- | --- |
| Neat1 | mmu-miR-377-3p, mmu-miR-107-3p, mmu-miR-185-5p, mmu-miR-31-5p |
| Mir17hg | mmu-miR-130a-3p |
