# Supplementary material for: Construction of a lncRNA-associated competing endogenous RNA regulatory network after traumatic brain injury in mouse
Source: Mol Brain. 2022 May 2;15:40. doi: 10.1186/s13041-022-00925-8 (PMC9063179; doi:10.1186/s13041-022-00925-8)
Supplement: Supplementary file 4 — Additional file 4. Interactions between miRNA and mRNA in the ceRNA network. [file 13041_2022_925_MOESM4_ESM.docx]

**Additional file 4. Interactions between miRNA and mRNA in the ceRNA network.**

| miRNA | mRNA |
| --- | --- |
| mmu-miR-107-3p | Cdk6 |
| mmu-miR-130a-3p | Rhod, Irf1 |
| mmu-miR-185-5p | Tead1, Ifi203, Vav1 |
| mmu-miR-31-5p | Myd88, Krt16, Krt17, Stk40 |
| mmu-miR-377-3p | Ifit3, Tnfrsf1b, Prrg4, Igfbp3, Ehf, Itgam, Pappa, Cd1d1, Sele, Adamts9, Clec4a2, Ifit3b, Spp1 |
